# Study on carotid artery stenosis after radiotherapy for nasopharyngeal carcinoma

**DOI:** 10.1007/s00432-024-05788-1

**Published:** 2024-05-25

**Authors:** JunMei Song, Luo Lan, YuQing Lv, YaJing Wen, Min Kang, RenSheng Wang

**Affiliations:** 1 Department of Radiation Oncology, The First Affiliated Hospital of Guangxi Medical University, Nanning, 530021 Guangxi China; 2 Oncology Department, Nanchong Hospital, The Second Clinical Institute of North Sichuan Medical College, Nanchong, 637000 Sichuan China; 3 Ministry of Education, Laboratory of Early Prevention and Treatment for Regional High Frequency Tumor (Guangxi Medical University), Nanning, 530021 China; 4 Nanning First People’s Hospital, Nanning, 530016 Guangxi China; 5 Epartment of Radiation Oncology, Nanfang Hospital, Southern Medical University, Guangzhou, 510515 China

**Keywords:** Nasopharyngeal carcinoma, Radiation therapy, Carotid stenosis, Ultrasonography

## Abstract

**Objective:**

This study investigated carotid artery stenosis (CAS) and associated risk factors in patients with nasopharyngeal carcinoma (NPC) post-radiotherapy.

**Materials and methods:**

The observation group comprised 86 reexamined patients with NPC, divided into Group 1 and Group 2 based on post-radiotherapy duration, alongside 34 newly diagnosed patients with NPC (Group 0). Carotid artery ultrasonography and chi-square analysis were performed.

**Results:**

Moderate-to-severe vascular abnormalities were exclusively in Group 2. Considering mild vascular abnormalities as the standard, the overall vascular abnormality rates in Group 2 and Group 0 were 65.9% and 41.2%, respectively. In Group 2 and Group 0, the abnormality rates for unilateral carotid artery (UCA), common carotid artery (CCA), internal carotid artery (ICA), and external carotid artery (ECA) were 47.4% and 30.9%, 44.3% and 22.1%, 44.3% and 16.2%, and 39.8% and 5.9%, respectively. Comparing group 1 to group 0, only UCA abnormalities were statistically significant (45.4% vs. 30.9%). Considering moderate-to-severe vascular abnormalities as the standard, Group 2 had higher overall vascular, UCA, CCA, ICA, and ECA abnormality rates compared to Group 0. The age at revisit over 45 years, T stage, and N stage may influence CAS.

**Conclusion:**

Radiation increasing CAS incidence after 3 years. So, regular examinations are recommended to dynamically monitor CAS after 3 years of radiotherapy.

## Introduction

Nasopharyngeal carcinoma (NPC) is a distinct malignancy of the head and neck that primarily originates in the nasopharyngeal mucosal epithelium. NPC exhibits pronounced regional disparities in occurrence, with Southern China reporting the highest global incidence rate, reaching 30–50 cases per 100,000 individuals (Chang and Adami 2006; Torre et al. 2015; Mahdavifar et al. 2016). In China, more than 90% of NPC cases manifest as undifferentiated non-keratinized squamous cell carcinoma, a subtype that demonstrates relative sensitivity to radiation therapy. Given its unique anatomical location and biological behavior, radiation therapy remains the principal therapeutic modality for NPC (Chen et al. 2018). Owing to their proximity to the lymph nodes of the neck and primary tumors of the nasopharynx, cervical vessels are frequently exposed to high levels of radiation, which can result in damage. Following an injury to the exposed blood vessels, progressive intimal medial thickening (IMT) occurs, leading to the development of atherosclerosis and carotid artery stenosis (CAS). Once CAS reaches a certain stage, individuals may experience transient syncope, transient ischemic attacks (TIA), and cerebral infarction. Consequently, the occurrence of TIA is closely associated with CAS (Venkatesulu et al. 2018). The advent of intensity-modulated radiation therapy (IMRT) has significantly improved the 5-year overall survival rate of patients with NPC to 80% (Lee et al. 2005; Peng et al. 2017). Consequently, investigation of carotid artery injury following radiotherapy has gained increasing research significance. Our focus now lies on enhancing the quality of life, minimizing radiotherapy-related complications, and ameliorating the late side effects associated with radiotherapy. Consequently, the clinical significance of early carotid artery screening after radiotherapy in patients with NPC has garnered increasing attention.

Several studies have provided evidence that radiotherapy is an independent risk factor for stroke (Dorresteijn et al. 2002; Halak et al. 2002). Chang et al. (2009) demonstrated a significant increase in the occurrence of bilateral arterial plaques among patients who received radiotherapy compared to those who did not. Brown et al. (2005) reported a significant increase in the incidence of CAS 10–15 years after radiotherapy (*P* = 0.03). Additionally, Cheng et al. (2000) found that patients who underwent radiotherapy more than 5 years ago had a relative risk of 15 for CAS and a relative risk of 38.5 for stroke or TIA. One study revealed that the prevalence of significant CAS exceeding 50% in patients with NPC after 3 years of radiotherapy may reach 23% (Greco et al. 2012). Numerous studies have consistently demonstrated that as the arterial intima thickens, the likelihood of CAS increases, subsequently increasing the risk of stroke (Muzaffar et al. 2000; Han et al. 2020). Consequently, there is growing emphasis on timely arterial screening in patients. Furthermore, apart from the duration following radiotherapy, the radiotherapy dosage is also a significant factor. However, the effect of irradiation dosage on blood vessels in patients with NPC remains inconclusive. Some studies suggest that the extent of vascular damage increases with increasing dosage (Dorth et al. 2014), whereas others propose the existence of a specific dosage threshold for radiotherapy-induced vascular injury with no observable dosage effect within the therapeutic range (Martin et al. 2005). The existing literature highlights the association between NPC radiotherapy and carotid artery injury, emphasizing the increased risk of stroke. However, there is a notable gap in understanding the specific correlation between the degree of carotid artery injury, the time interval following radiotherapy, and the dosage of radiotherapy in patients with NPC. Additionally, the literature does not provide conclusive evidence on the impact of irradiation dosage on blood vessels in NPC patients.

This study employed cervical blood vessel color Doppler ultrasound to observe carotid artery injury in all participants and subsequently examined the correlation between the degree of carotid artery injury in patients with NPC after radiotherapy, time interval after radiotherapy, and dosage of radiotherapy. Additionally, potential factors influencing the degree of CAS after radiotherapy were analyzed. Aims to provide a more nuanced and comprehensive understanding of the factors contributing to vascular damage. Aid clinicians in assessing and managing the risks associated with carotid artery injury more effectively.

## Materials and methods

### Participants

Inclusion criteria (observation group): (1) Patients with NPC who had previously received radiotherapy and revisited the First Affiliated Hospital of Guangxi Medical University between September 2020 and January 2021. (2) Nasopharyngeal squamous cell carcinoma confirmed by pathological examination. (3) Patients who completed the entire course of radiotherapy and had complete radiotherapy plan data. Exclusion criteria: (1) Recurrence of primary lesions and the cervical lymphatic drainage area. (2) History of head and neck radiotherapy or surgery prior to radiotherapy. (3) The occurrence of cardiovascular and cerebrovascular events prior to radiotherapy. This study was approved by the Institutional Review Committee of the First Affiliated Hospital of Guangxi Medical University.

Inclusion criteria (control group): (1) Patients diagnosed with NPC who were initially treated at the First Affiliated Hospital of Guangxi Medical University between September 2020 and January 2021. (2) No administration of treatment before the cervical vascular examination. (3) Confirmation of nasopharyngeal squamous cell carcinoma by pathological examination. Exclusion criteria: (1) Individuals with a history of head and neck surgery or radiotherapy. (2) Occurrence of cardiovascular and cerebrovascular disease events in the past. (3) Individuals whose data collection process was significantly affected by cervical lymph node pressure.

The study group was divided into two subgroups based on the time interval after radiotherapy. Group 1 encompassed individuals who were ≥ 12 months but < 36 months post-radiotherapy, while Group 2 consisted of those who were ≥ 36 months post-radiotherapy (with time intervals less than 1 month being considered as 1 month). The control group was denoted as Group 0.

### Color ultrasonic Doppler examination

All enrolled patients were randomly examined by two experienced sonographers using a Mindray Resona7 color Doppler ultrasound diagnostic instrument. Throughout the examination, all patients were placed in the supine position, with the head extended to fully expose both sides of the neck. The probe was maneuvered from the proximal end of the common carotid artery (CCA) to the distal end of the transverse section. The entire CCA, bifurcation of the internal carotid artery (ICA), a distance of 4–6 cm beyond it, and the trunk of the external carotid artery (ECA) were continuously observed. The vascular intima and flow rates were observed in the transverse and longitudinal sections.

### Detection index and grading standard

The indicators for detection included the vascular diameter, IMT, peak systolic velocity (PSV, cm/s), and end-diastolic velocity (EDV, cm/s) of the vessels in the bilateral CCA, ICA, and ECA. Additionally, the proportion of diameter stenosis or area stenosis was considered when necessary. According to the Chinese Guidelines for Vascular Ultrasound Examination of Stroke developed by the Stroke Prevention and Treatment Engineering Committee of the National Health and Family Planning Commission (Hua et al. 2015), the thickening of the carotid internal media-membrane is defined as IMT ≥ 1.00 mm, while IMT ≥ 1.50 mm or intima thickening protruding from the lumen or local intima thickness higher than 50% of peripheral intima thickness indicates the formation of atherosclerotic plaque. The degree of CAS is based on plaque, flow rate, and pipe diameter. ICA can be categorized into four levels based on these criteria (Appendix 1). The evaluation of CCA and ECA stenosis cannot be determined based on the flow rate according to the norms since the ICA blood flow ratio, assessed through PSV_ICA_/PSV_CCA_, is used to measure the degree of stenosis. Instead, the stenosis condition is calculated by considering the proportion of tube diameter stenosis or area stenosis in accordance with the classification of the ICA vascular stenosis degree.

In this study, intimal thickening and mild stenosis (< 50%) were defined as mild vascular abnormalities, moderate stenosis (50% ≤ stenosis < 70%) as moderate vascular abnormalities, and severe (≥ 70%) stenosis as severe vascular abnormalities.

### Treatment

All patients in the observation group received IMRT, either in combination with concurrent radiochemotherapy (CCRT), induction chemotherapy (IC), or adjuvant chemotherapy (AC). The prescribed dose for the PGTVnx was 68–74 Gy. The PTVnd was 64–70 Gy, PTV1 was 60–64 Gy, and PTV2 was 54–56 Gy, administered five times per week, totaling 31–33 times.

Among the chemotherapy regimens, TP (docetaxel 75 mg/m^2^ and cisplatin 80–100 mg/m^2^ every three weeks) was predominantly used in the IC and AC protocols. In the concurrent chemotherapy regimen, cisplatin was administered at a dosage of 80–100 mg/m^2^ every three weeks or 30–40 mg/m^2^ weekly for a total of 2–3 cycles.

### Carotid target area delineation and dose classification

The treatment plan for the patients was obtained from the planning system, and the same radiotherapy physician involved in NPC treatment outlined the target areas of the CCA, ICA, and ECA for the patients in the observation group. The delineation of the arteries extending from the skull base to the clavicle level (Carpenter et al. 2018), and the maximum dose (Dmax) of each blood vessel was recorded using a dose-volume histogram (DVH) diagram. The median Dmax dose was established as the threshold value, with arteries receiving a dose lower than the threshold classified as the low-dose group and those receiving a dose equal to or greater than the threshold classified as the high-dose group.

### Statistics

Data analysis was conducted using SPSS version 23.0. Pearson's chi-square test, chi-square continuous correction test, (2 × C) chi-square test, and Fisher's exact test were utilized to compare different data types. Statistical significance was set at *P* < 0.05.

## Result

### General clinical data analysis

The study included 120 individuals, with 86 participants assigned to the observation group and 34 to the control group. The median time elapsed after radiotherapy for Group 1 and Group 2 was 1.8 years (range: 1–2.9 years) and 4.3 years (range: 3–6.9 years), respectively. No statistically significant differences were observed between the observation and control groups (Table 1).

**Table 1:** Comparison of general data between the observation group and control group

Variables	Group 0	Group 1	Group 2	P1	P2
	*n* = 34	*n* = 42	*n* = 44		
Sex				0.424	0.988
Male	27	30	35		
Female	7	12	9		
Age				0.456	0.626
<50	19	27	27		
≥ 50	15	15	17		
Hyperglycemia				1^a^	0.921^a^
No	32	40	40		
Yes	2	2	4		
Hyperlipidemia				0.279	0.762
No	22	22	27		
Yes	12	20	17		
Hypertension				1^a^	1^a^
No	32	39	41		
Yes	2	3	3		
Smoking				0.951	0.665
No	14	17	16		
Yes	20	25	28		
Histological type				1^a^	1^a^
WHO III	31	40	40		
WHO II	3	3	4		
T stage				0.403^b^	0.754^b^
T1 + 2	10	8	10		
T3	14	16	19		
T4	10	18	15		
N stage				0.98	0.665
N0 + 1	14	17	16		
N2 + 3	20	24	28		
M stage				1^a^	0.680^a^
M0	29	36	40		
M1 + X	5	5	4		

P1 represents the comparison between group 1 and group 0; P2 represents the comparison between group 2 and group 0

TNM staging followed the 8th edition of the American Joint Commission on Cancer (AJCC) staging system

*WHO* World Health Organization

^a^Chi-square continuous calibration test

^b^2 × C Chi-square test

### Reliability analysis of data acquisition

To assess the stability and reliability of the neck blood vessel data collection, the results from the two sonographers were compared for the initial 15 patients in the observation group. Blood vessels at all levels of the patient were considered as the unit, and mild vascular abnormalities were considered as the test standard, indicating that there was no statistical difference in the reliability of the test results between the two sonographers (Table 2) (*P* > 0.05).

**Table 2:** Comparison of test results of 15 patients in the observation group by two sonographers

	CCA (*N* = 30)			ICA (*N* = 30)			ECA (*N* = 30)		
Group	A	B	*P* = 0.789^a^	A	B	*P* = 1^a^	A	B	*P* = 1^a^
Normal	10	12		11	10		16	17	
Abnormal	20	18		19	20		14	13	

*CCA*, common carotid artery, *ICA* internal carotid artery, *ECA* external carotid artery

^a^Fisher's exact test

### Analysis using mild vascular abnormality as the standard

Considering potential multiple vascular injuries on one side of the same patient's blood vessel, comparisons were made between the rates of overall vascular abnormalities in the patient (Table 3), unilateral vascular abnormalities (Table 4), and vascular abnormalities at all levels (Table 5). When comparing Group 0 with Group 1, the overall vascular abnormality rate was 41.2% vs. 59.5% (*P* = 0.112). The rates of unilateral carotid artery (UCA) abnormalities were 30.9% vs. 45.4% (*P* = 0.001). Additionally, the rates of mild vascular abnormalities at all levels were as follows: CCA: 22.1% vs. 33.3% (*P* = 0.125); ICA: 16.2% vs. 28.6% (*P* = 0.071); and ECA: 5.9% vs. 15.5% (*P* = 0.062). When comparing Group 0 with Group 2, the overall vascular abnormality rate was 41.2% vs. 65.9% (*P* = 0.029). The rates of UCA abnormalities were 30.9% vs. 47.4% (*P* < 0.001). Additionally, the mild vascular abnormalities rate at all levels was as follows: CCA: 22.1% vs. 44.3% (P = 0.004); ICA: 16.2% vs. 44.3% (*P* < 0.001); and ECA: 5.9% vs. 39.8% (*P* < 0.001). The findings of this study demonstrated significant statistical differences in the rates of overall vascular abnormalities, unilateral vascular abnormalities, and all levels of vascular abnormalities after 3 years of radiotherapy compared with patients who did not receive radiotherapy. Additionally, a statistically significant difference was observed in the occurrence of unilateral mild vascular abnormalities 1 year after radiotherapy compared to patients who did not receive radiotherapy.

**Table 3:** Comparison of overall mild vascular abnormality rates between the observation and control group

Group	Group 0 (*N* = 34)	Group 1 (*N* = 42)	Group 2 (*N* = 44)	P1	P2
Normal	20 (58.8%)	17 (40.5%)	15 (34.1%)	0.112	0.029^*^
Abnormal	14 (41.2%)	25 (59.5%)	29 (65.9%)		

P1 represents the comparison between group 1 and group 0; P2 represents the comparison between group 2 and group 0

^*^Statistically significant

**Table 4:** Comparison of mild abnormal rate of unilateral vessels between the observation group and the control group

Group	Group 0 (*N *= 68)	Group 1 (*N* = 84)	Group 2 (*N* = 88)	P1	P2
Normal	47 (69.1%)	36 (54.6%)	35 (52.6%)	0.001^*^	<0.001^*^
Abnormal	21 (30.9%)	48 (45.4%)	53 (47.4%)		

P1 represents the comparison between group 1 and group 0; P2 represents the comparison between group 2 and group 0

^*^Statistically significant

**Table 5:** Comparison of mild vascular abnormality rates at all levels between the observation group and the control group

		CCA				ICA				ECA		
	Group 0	Group1	Group2		Group 0	Group1	Group 2		Group 0	Group 1	Group2	
	*N* = 68	*N* = 84	*N* = 88		*N* = 68	*N* = 84	*N* = 88		*N* = 68	*N* = 84	*N* = 88	
Normal	53	56	49	P1 = 0.125	57	60	49	P1 = 0.071	64	71	57	P1 = 0.062
	77.9%	66.7%	55.7%		83.8%	71.4%	55.7%		94.1%	84.5%	60.2%	
Abnormal	15	28	39	P2 = 0.004*	11	24	39	P2 < 0.001*	4	13	35	P2 < 0.001*
	22.1%	33.3%	44.3%		16.2%	28.6%	44.3%		5.9%	15.5%	39.8%	

P1 represents the comparison between group 1 and group 0; P2 represents the comparison between group 2 and group 0

*CCA* common carotid artery, *ICA* internal carotid artery, *ECA* external carotid artery

^*^Statistically significant

### Dose analysis using mild vascular abnormality as the standard

Vascular injury is primarily influenced by the hotspot dose. To assess the impact of the radiotherapy dose on blood vessels, the median Dmax dose of all blood vessel levels in all observation groups was used as the threshold value, and the blood vessels were categorized into high-dose and low-dose groups. The median CCA dose was 71.4 Gy (59.6–77.5 Gy), the ICA dose was 75.1 Gy (58.8–77.7 Gy), and the ECA dose was 72.6 Gy (60–77.6 Gy). The incidence rates of vascular abnormalities in the low-dose and high-dose groups were as follows: CCA: 17.4% vs. 24.4% (*P* = 0.261); ICA: 17.4% vs. 18.6% (*P* = 0.843); and ECA: 7.0% vs. 12.8% (*P* = 0.201). The results indicated that there were no differences in mild vascular abnormalities in the neck between the two groups (Table 6).

**Table 6:** Comparison of effects of dose on mild vascular abnormalities after radiotherapy

Group	CCA	(*N* = 172)		ICA	(*N* = 172)		ECA	(*N* = 172)	
	Normal	Abnormal	*P* = 0.261	Normal	Abnormal	*P* = 0.843	Normal	Abnormal	*P* = 0.201
Low	71	15		71	15		80	6	
	82.6%	17.4%		82.6%	17.4%		93.0%	7.0%	
High	65	21		70	16		75	11	
	75.6%	24.4%		81.4%	18.6%		87.2%	12.8%	

*CCA* common carotid artery, *ICA* internal carotid artery, *ECA* external carotid artery

### Analysis using moderate and severe vascular abnormalities as the standard

Moderate or higher vascular abnormalities occurred only in group 2, among which one had severe stenosis, and 18 had moderate vascular abnormalities. No TIA occurred in any patient. The overall rates of moderate and severe vascular abnormalities in Group 2 and Group 0 were 43.2% vs. 0% (*P* < 0.001). The rate of unilateral vascular abnormalities was 26.14% vs. 0% (*P* < 0.001). The rate of vascular abnormalities at all levels was as follows: CCA: 31.8% vs. 0% (*P* < 0.001); ICA: 29.5% vs. 0% (*P* < 0.001); and ECA: 31.8% vs. 0% (*P* < 0.001). These findings suggest that patients who received radiotherapy exhibited a higher prevalence of moderate and severe vascular abnormalities 3 years post-radiotherapy than those who did not receive radiotherapy (Table 7).

**Table 7:** Comparison of moderate vascular abnormalities at all levels between group 2 and control group

Group	CCA			ICA			ECA		
	Group 0 *N* = 68	Group 2 *N* = 88		Group 0 *N* = 68	Group 2 *N* = 88		Group 0 *N* = 68	Group 2 *N* = 88	
Normal	68	60	*P* < 0.001^*^	68	62	*P* < 0.001^*^	68	60	*P* < 0.001^*^
	100%	68.2%		100%	70.5%		100%	68.2%	
Abnormal	0	28		0	26		0	28	
	0%	31.8%		0%	29.5%		0%	31.8%	

*CCA* common carotid artery, *ICA* internal carotid artery, *ECA* external carotid artery

^*^Statistically significant

### Dose analysis was performed with moderate and severe vascular abnormalities as the standard

The occurrences of moderate and severe vascular abnormalities in the low- and high-dose groups were as follows: CCA, 6.8% vs. 15.9% (*P* = 0.179); ICA, 11.4% vs. 18.2% (*P* = 0.367); and ECA, 4.5% vs. 11.4% (*P* = 0.431). The results indicated no significant difference in moderate and severe vascular abnormalities between the two groups (*P* > 0.05) (Table 8).

**Table 8:** Comparison of the effects of dose on moderate vascular abnormalities in patients 3 years after radiotherapy

Group	CCA (*N* = 88)			ICA (*N* = 88)			ECA (*N* = 88)		
	Normal	Abnormal	*P* = 0.179	Normal	Abnormal	*P* = 0.367	Normal	Abnormal	*P* = 0.431^a^
Low	41	3		39	5		42	2	
	93.2%	6.8%		88.6%	11.4%		95.5%	4.5%	
High	37	7		36	8		39	5	
	84.1%	15.9%		81.8%	18.2%		88.6%	11.4%	

*CCA* common carotid artery, *ICA *internal carotid artery, *ECA* external carotid artery

^a^ Chi-square continuous calibration test

### Analysis of influencing factors of moderate and severe vascular abnormalities after radiotherapy

Considering the clinical importance of moderate or high vascular stenosis in practice, patients in Group 2 were categorized into normal and abnormal groups based on their vascular conditions. Both groups were examined for general conditions, treatment factors, and factors affecting traditional atherosclerosis. The findings revealed that the age at revisit over 45 years, T stage, and N stage were significant influencing factors (Table 9).

**Table 9:** The influences of various factors on moderate and severe CAS were analyze

Characteristic	Normal	A*bnormal *	*P*
Age^#^			0.040^a^
≤ 45	17 (68.0%)	7 (36.8%)	
>45	8 (32.0%)	12 (63.2%)	
Age when radiotherapy			0.533
≤ 45	18 (72.0%)	12 (63.2%)	
>45	7 (28.0%)	7 (36.8%)	
Pathological pattern			0.178
WHO III	24 (96.0%)	16 (84.2%)	
WHO II	1 (4.0%)	3 (15.8%)	
Time^*^			0.062
<52 month	15 (60.0%)	6 (31.6%)	
≥ 52 month	10 (40.0%)	13 (68.4%)	
Hyperlipidemia			0.096
No	18 (72.0%)	9 (47.4%)	
Yes	7 (28.0%)	10 (52.6%)	
T stage			0.041^a^
T1 + 2	9 (36.0%	1 (5.3%)	
T3 + 4	16 (64.0%)	18 (94.7%)	
N stage			0.013^a^
N0 + 1	13 (52.0%)	3 (15.8%)	
N2 + 3	12 (48.0%)	16 (84.2%)	
M stage			0.061^b^
M0	25 (100%)	15 (78.9%)	
M1 + x	0 (0%)	4 (21.1%)	
Smoking			0.954
No	9 (36.0%)	7 (36.8%)	
Yes	16 (64.0%)	12 (63.2%)	
Diabetes			0.498^c^
No	23 (92.0%)	19 (100%)	
Yes	2 (8.0%)	0 (0%)	
Hypertension			0.146^b^
No	25 (100%)	16 (84.2%)	
Yes	0 (0%)	3 (15.8%)	
CCA			0.179
High dose radiotherapy	37 (84.1%)	7 (15.9%)	
Low dose radiotherapy	41 (93.2%)	3 (6.8%)	
ICA			0.367
High dose radiotherapy	36 (81.8%)	8 (18.2%)	
Low dose radiotherapy	39 (88.6%)	5 (11.4%)	
ECA			0.431^b^
High dose radiotherapy	39 (88.6%)	5 (11.4%)	
Low dose radiotherapy	42 (95.5%)	2 (4.5%)	

TNM staging followed the 8th edition of the American Joint Commission on Cancer (AJCC) staging system

*CCA* common carotid artery, *ICA* internal carotid artery, *ECA* external carotid artery, *WHO *World Health Organization

^#^The age at revisited

^*^Interval between examination and radiotherapy

^a^*P*<0.05

^b^Chi-square continuous calibration test

^c^Fisher's exact test

## Discussion

NPC is a common malignant tumor in southern China, with 75-87% of patients with NPC exhibiting lymph node metastasis at initial diagnosis (Ho et al. 2012; Li et al. 2013). In the course of radical radiotherapy for head and neck tumors, the lymphatic drainage area of the neck is exposed to high radiation doses, potentially resulting in radiation-induced damage to the blood vessels of the neck. With advancements in treatment techniques, the 5-year survival rate of patients with NPC has improved. This increased survival rate correlates with a heightened history of vascular injury after radiotherapy, making the impact of CAS on patients' long-term quality of life more obvious.

Currently, the pathophysiological mechanisms underlying radiation-induced vascular injury remain unclear. It is widely accepted among researchers that radiation primarily damages the vascular endothelial cells and vascular fiber tissues (Hatoum et al. 2006; Plummer et al. 2011; Gujral et al. 2014; Simonetti et al. 2014). Investigations have revealed that the extent and length of carotid artery injury following radiotherapy surpass those observed in traditional atherosclerosis and exhibit an atypical distribution (Lam et al. 2002; Shichita et al. 2009). Considering the mechanism of vascular radiation damage, the damage mode is categorized as a late-response damage mode, and its occurrence and development must be a long-term process.

Based on existing research, the temporal progression of CAS remains uncertain. Various studies have reported different incidence rates of CAS at different time points. Dorresteijn et al. (2002) discovered that patients who underwent radiotherapy experienced initial symptoms such as cerebral ischemia anywhere between 6 months and 20 years after treatment. Similarly, Greco et al. (2012) observed a higher occurrence of moderate and severe stenosis progression after 3 years in patients with head and neck tumors who underwent both surgery and radiotherapy than in those who underwent surgery only (*P* < 0.05).

Lam et al. (2001) found that the overall carotid artery abnormality rate in a sample of 71 patients who had undergone radiotherapy 4–20 years prior was 78.9%. Furthermore, the incidences of CCA (77.5% vs. 21.6%), ECA (45% vs. 2%) and ICA (77.5% vs. 21.6%) lesions were significantly higher in the radiotherapy group than in the initial treatment group (*P* < 0.01). Similarly, Steele et al. (2004) conducted a prospective study involving 40 patients with head and neck tumors who received radiotherapy. Ultrasonography was used to monitor the patients over a median follow-up period of 10.2 years. The study revealed a 40% (16/40) incidence of significant carotid artery stenosis, with six patients experiencing vascular occlusion and three patients suffering from stroke. This finding suggests that the patient experienced notable vascular stenosis before the median follow-up period, thus necessitating the recommendation of noninvasive vascular screening for patients undergoing radiotherapy for head and neck tumors. However, the specific timing of the screening was not specified. Previous research has indicated that IMT can significantly increase 1 year after radiotherapy and continues to progress over time (Han et al. 2020). Huang et al. (2013) identified a critical time point of 42.5 months (3.5 years) post-radiotherapy for predicting the occurrence of carotid plaque.

In most previous studies, a single study group and a control group were employed for comparative analysis. The distinction in our study lies in the selection of one control group, which was then compared with two groups of patients: one group was assessed 1–3 years after radiotherapy, and another group was evaluated 3 years after radiotherapy. Vascular abnormalities were categorized as none, mild, or moderate. Notably, when examining mild vascular abnormalities, the rates of overall vascular abnormalities, unilateral vascular abnormalities, and all levels of vascular abnormalities in patients were statistically significant when compared between Group 2 and Group 0. There were significant differences in the rate of unilateral vascular abnormalities between Groups 1 and 0. Although there were no statistical differences between Group 1 and Group 0 in the overall rate of vascular abnormalities and the rate of all levels of vascular abnormalities, the *P* value was close to 0.05, and there were statistical differences in the comparison indicators between Groups 2 and 0, reflecting the development trend of vascular abnormalities over time. When moderate vascular abnormalities were considered as the standard, the rates of moderate vascular abnormalities, unilateral vascular abnormalities, or vascular abnormalities at all levels were statistically significant (*P* < 0.05). These aberrant results were consistent with the findings of Greco et al. (2012).

In addition to the radiation itself and the time interval after radiotherapy, the radiotherapy dose may also affect CAS. However, the findings from various studies have diverged on this matter. Previous studies have shown that radiation injury to large and medium blood vessels has a dose-dependent effect (Dorth et al. 2014). Conversely, other studies have demonstrated that the dose of vascular damage caused by radiation has a certain threshold and that there is no dose effect within the dose range in patients with head and neck tumors receiving radiation (Martin et al. 2005). Steele et al. did not observe a definitive correlation between cumulative radiotherapy dose and CAS severity (Steele et al. 2004). Previous investigations examining the impact of the radiotherapy dose on vascular injury in large and medium arteries within the standard therapeutic dose range for head and neck treatment using conventional or IMRT have yielded inconclusive results (Cheng et al. 2000; Li et al. 2010; Huang et al. 2013; Zhou et al. 2015). The current study also failed to establish a correlation between the radiation dose and vascular injury. The limited sample size or delayed response of the blood vessel itself may have accounted for the failure to observe a dose-dependent effect within the designated observation period. Prolonged follow-up is necessary to investigate the time-dependent nature of carotid artery lesions. Furthermore, the effect of traditional atherosclerotic factors on CAS after radiotherapy remains unclear. Lam et al. (2001) determined that there was no significant association between post-radiotherapy carotid artery injury and conventional factors influencing atherosclerosis, whereas Dorresteijn et al. (2002) established that hypertension was associated with an increased risk of ischemic stroke in patients after radiotherapy. The results of our study indicated that carotid artery injury is associated with the existing age, T stage, and N stage. The *P* value of the impact of blood lipid levels and time elapsed after radiotherapy on CAS was found to be very close to 0.05, which is potentially attributable to the limited sample size. The risk factors for radiation therapy cannot be altered in patients with NPC. However, conventional atherosclerotic factors and the duration of radiation therapy can be promptly identified and addressed through regular monitoring. Consequently, patients with NPC should undergo carotid artery ultrasound screening 3 years after radiotherapy, and even if there is no obvious CAS, the development trend can be predicted by intima thickening and plaque status.

This study had certain limitations. Despite the use of cross-sectional studies commonly employed in this field to compare diverse patient groups and eliminate numerous factors, there still exists a degree of heterogeneity among patients that cannot be disregarded. In the selection of the examination method, various factors such as convenience, affordability, safety, and repeatability were considered, leading to a preference for ultrasound examination over more accurate alternatives such as Magnetic Resonance Angiography (MRA) or Digital subtraction angiography (DSA). Consequently, the examination results exhibited a certain number of false-negative outcomes. In future study, we could employ more advanced imaging techniques like MRA or DSA to enhance accuracy in detecting vascular abnormalities.

## Conclusions

The rate of carotid artery abnormalities in patients 3 years after radiotherapy was higher than that in patients without radiotherapy. The age at revisit over 45 years, T stage, and N stage may be risk factors for CAS after radiotherapy. Therefore, regular examinations are recommended for NPC patients to dynamically monitor CAS after 3 years of radiotherapy.

## Figures and Tables

**Fig. 1:**
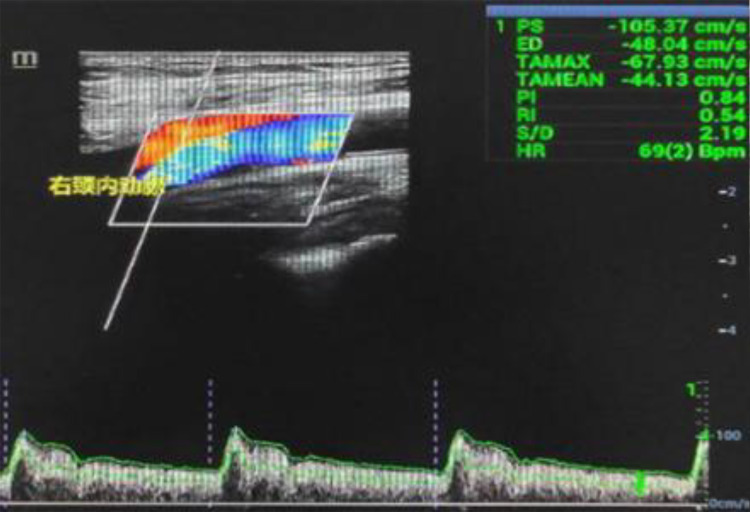
Shows the measurement of blood flow velocity on the longitudinal section of the vessel

**Fig. 2:**
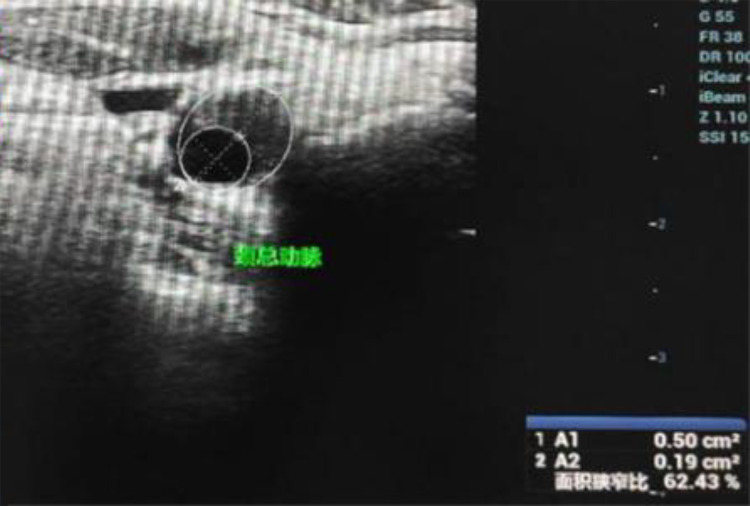
Shows the proportion of cross-sectional area to detect vascular abnormalities

**Fig. 3:**
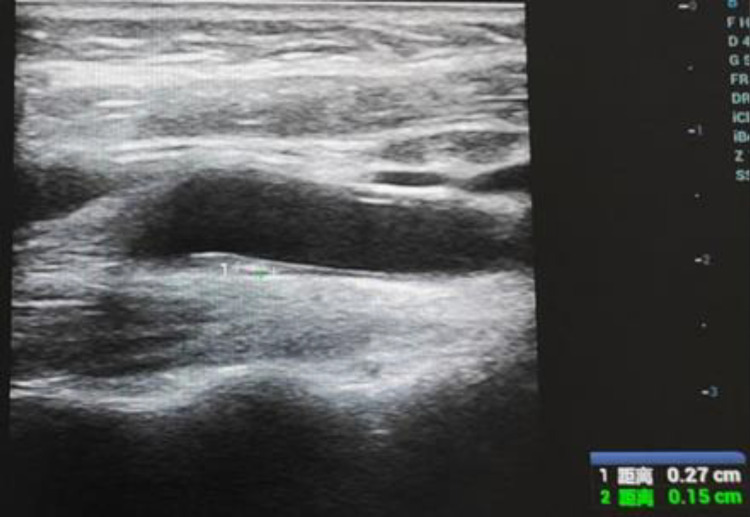
Shows the plaque formation in the longitudinal section of the vessel

**Fig. 4:**
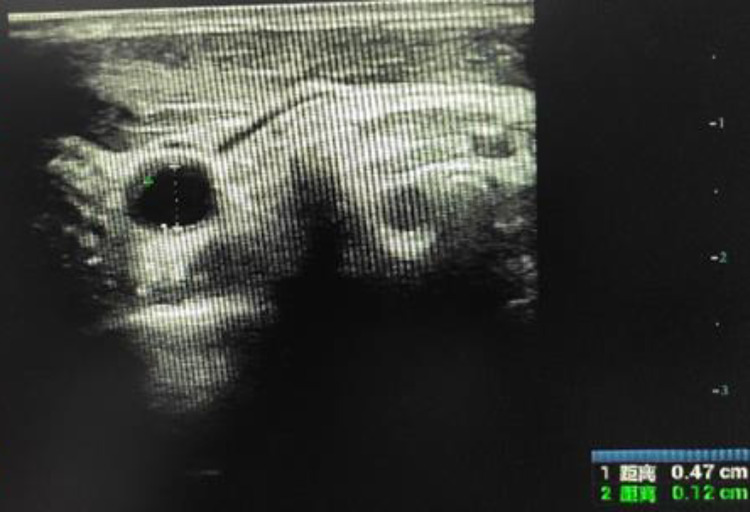
Shows the internal diameter measurement and plaque formation in the transverse section of blood vessels respectively

## Data Availability

Data for this study are available from the corresponding author upon reasonable request.

## Appendix 1

See Table 10

**Table 10:** Criteria for the degree of carotid stenosis

Stenosis/velocity	Intracaluminal	PSV (cm/s)	EDV (cm/s)	PSV_ICA_/PSV_CCA_
< 50% (0–49%)	With or without plaques	< 125	< 40	< 2.0
50–69%	plaque	≥ 125, < 230	≥ 40, < 100	≥ 2.0, < 4.0
70–99%	plaque	≥ 230	≥ 100	≥ 4.0
occlusion	Plaque or thrombus	No blood flow signal	No blood flow signal	No blood flow signal

## Appendix 2

See Figs. 1, 2, 3, 4
